# Rapid Detection of *Klebsiella pneumoniae* Carrying Virulence Gene *rmpA2* by Recombinase Polymerase Amplification Combined With Lateral Flow Strips

**DOI:** 10.3389/fcimb.2022.877649

**Published:** 2022-05-19

**Authors:** Na Li, Lei Wang, Fang Wang, Huimin Chen, Shuan Tao, Qing Zhu, Liping Liu, Wei Liang, Fang Ma

**Affiliations:** ^1^Department of Laboratory Medicine, The Second People’s Hospital of Lianyungang City, Affiliated to Bengbu Medical College, Lianyungang, China; ^2^Department of Medical Laboratory, Bengbu Medical College, Bengbu, China; ^3^School of Biotechnology, Jiangsu University of Science and Technology, Zhenjiang, China; ^4^Department of Central Laboratory, Lianyungang Second People’s Hospital affiliated to Jiangsu University, Lianyungang, China; ^5^Lianyungang Second People’s Hospital affiliated to Xuzhou Medical University, Lianyungang, China

**Keywords:** highly virulent *Klebsiella pneumoniae*, recombinase polymerase amplification, rapid test, *rmpA2*, base substitution, lateral flow strip (LFS)

## Abstract

Highly virulent *Klebsiella pneumoniae* often causes invasive infections with high morbidity and mortality rates, posing an immense clinical challenge. Rapid and accurate detection of pathogenic bacteria is of great significance for treatment and preventive control. Conventional detection by polymerase chain reaction (PCR) is limited by a dependence on laboratory equipment and professional staff. Recombinase polymerase amplification (RPA) combined with a lateral flow strip (LFS) can rapidly amplify and visualize target genes in a short period of time. The aim of this study was to develop an RPA-LFS technique for detection of the *K. pneumoniae* virulence gene *rmpA2*. Primers were designed against conserved sequences specific to the virulence gene, and primer probe design was optimized by introducing base substitution to obtain a specific and sensitive primer-probe combination for clinical detection. We tested 65 actual samples collected from clinics to evaluate the performance of the newly established RPA-LFS system in comparison with conventional PCR methods and qPCR methods. The RPA-LFS assay was performed at for 25 min a constant temperature of 37°C, and results could be observed without instrumentation. The system could specifically identify highly virulent *K. pneumoniae* carrying the virulence gene *rmpA2* with a minimum detection limit of 10^−1^ ng/μL and 10 copies/μL. For the 65 clinical samples tested, The RPA-LFS assay results were in complete agreement with the qPCR results and PCR results. The RPA-LFS assay provides a rapid, accurate, and simple method for identification of highly virulent *K. pneumoniae* carrying *rmpA2*.

## Introduction

*Klebsiella pneumoniae* is a common causative agent of hospital-acquired infections occurring in patients with multiple comorbidities in hospitals or long-term care facilities (Shon and Russo, 2012). *K. pneumoniae* strains can be divided into two categories based on their mucoid nature: classical *K. pneumoniae* (cKP)and hypervirulent *K. pneumoniae* (hvKP). The first case of liver abscess combined with infectious endophthalmitis due to highly virulent *K. pneumoniae* infection was reported in Taiwan, China, in 1986, and eventually led to blindness in most patients despite aggressive treatment with numerous antibiotics (Liu et al., 1986). hvKP mainly causes invasive infections, such as bloodstream infections and purulent liver abscesses, which mainly infect young, immunocompetent individuals. hvKP virulence factors include podoconjugate, Fimbriae, lipopolysaccharide, and iron uptake (Siu et al., 2012). These features contribute to its unique pathogenic capacity.

**Figure 1:**
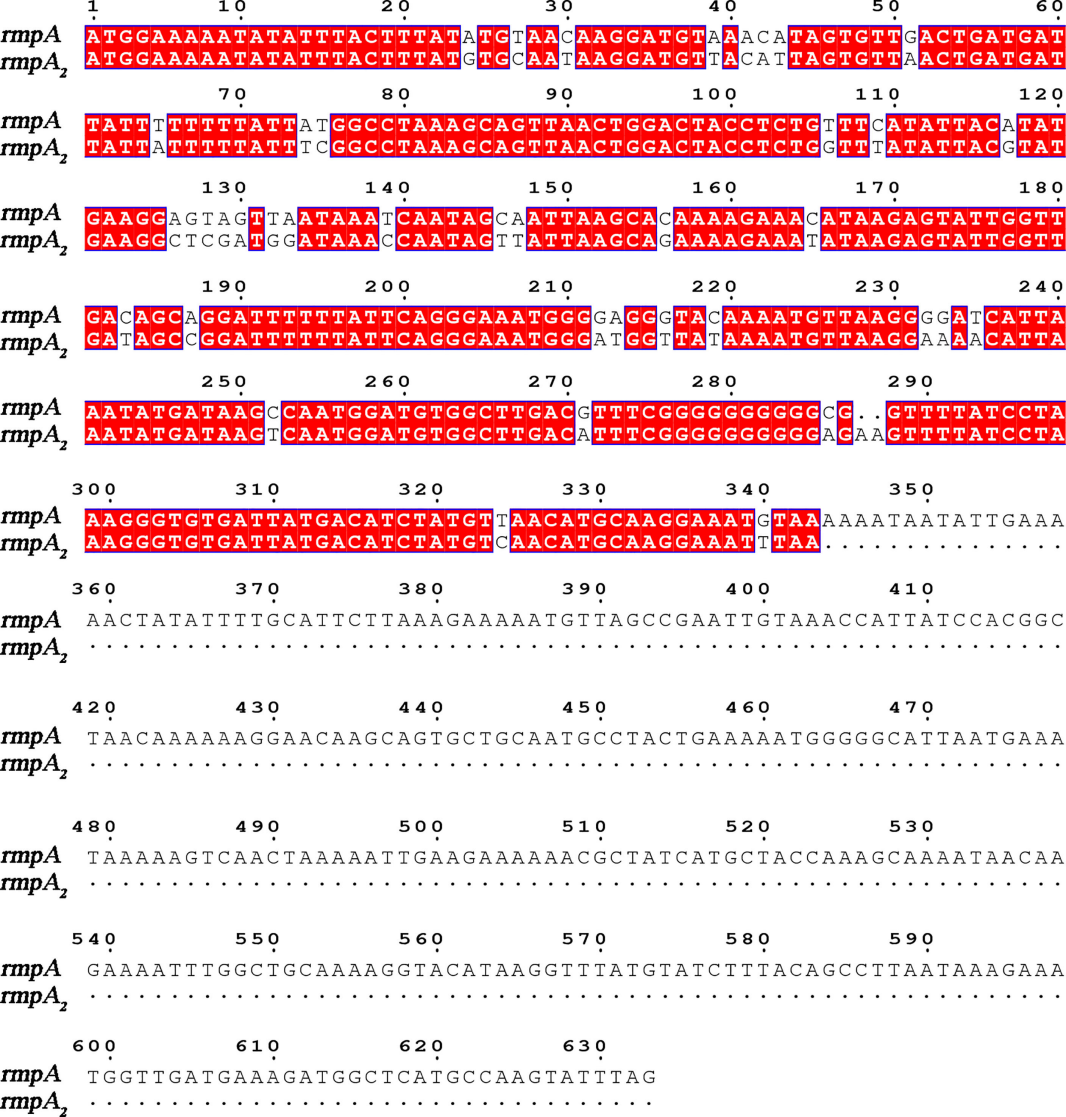
Sequence alignment of rmpA2 and rmpA genes.

There is no accepted definition of hvKP to date. The mouse infection assay is currently the gold standard for identifying the virulence of *K. pneumoniae* (Yu et al., 2007). With the development of genomics, virulence plasmids such as pK2044, pLVPK, and pVir-CR-hvK4 have been identified in hvKP strains (Chen et al., 2004; Wu et al., 2009; Gu et al., 2018). Toxigenic plasmids can be spontaneously transferred between strains, and classical *K. pneumoniae* can be characterized as highly virulent due to the acquisition of virulence plasmids (Yang et al., 2019). The virulence factors causing the hypervirulence phenotype are encoded by genes present on these plasmids, including *iuc*, *peg-344*, *iro*, *rmpA*, and *rmpA2*. *rmpA*/*rmpA2* (*mucus phenotype regulatory gene A*) is important for mediating the expression of the hypervirulence phenotype, regulating the production of large amounts of podocytes (**Figure 1**) (Liu et al., 1986; Cheng et al., 1991; Fierer et al., 2011). Its expression increases podocyte production and enhances anti-macrophage phagocytosis, thereby enhancing the virulence of *K. pneumoniae* (Cheng et al., 2010). *K. pneumoniae* strains are divided into highly virulent and non-highly virulent groups based on the results of infection experiments in mice. The sensitivity of *rmpA2* assay was 88.89%, the specificity was 93.1%, the positive predictive value was 90.91%, and the negative predictive value was 91.53% (Li et al., 2020); therefore, detection of the virulence gene *rmpA2* can help to predict the virulence of *K. pneumoniae*.

Currently available assays for the virulence gene *rmpA2* employ polymerase chain reaction (PCR) and MALDI-TOF MS techniques (Yu et al., 2015). These techniques are time-consuming and laborious with high instrument dependence. PCR-based methods can give results in a few hours and have become the main method for pathogen detection (Salazar et al., 2015). However, the need for complex thermal cyclers and trained professionals limits their use in remote areas. By contrast, isothermal recombinant polymerase amplification (RPA) does not have these cumbersome requirements and can therefore be more widely used for rapid field assays (Daher et al., 2016; Dai et al., 2019).

**Figure 2:**
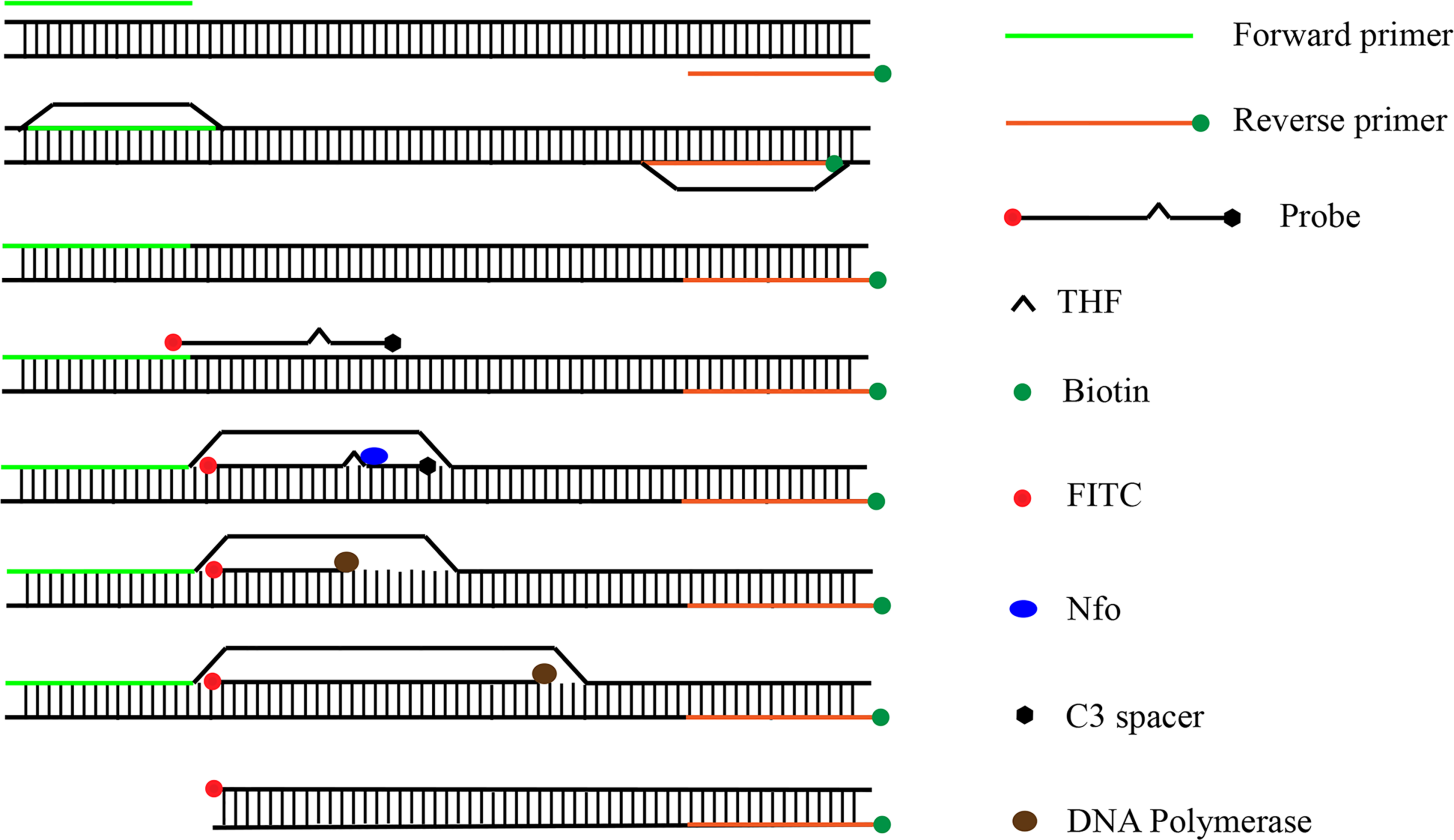
Amplification principle of RPA-LFS. The shapes and their representative molecules are listed on the right side of the drawing.

**Figure 3:**
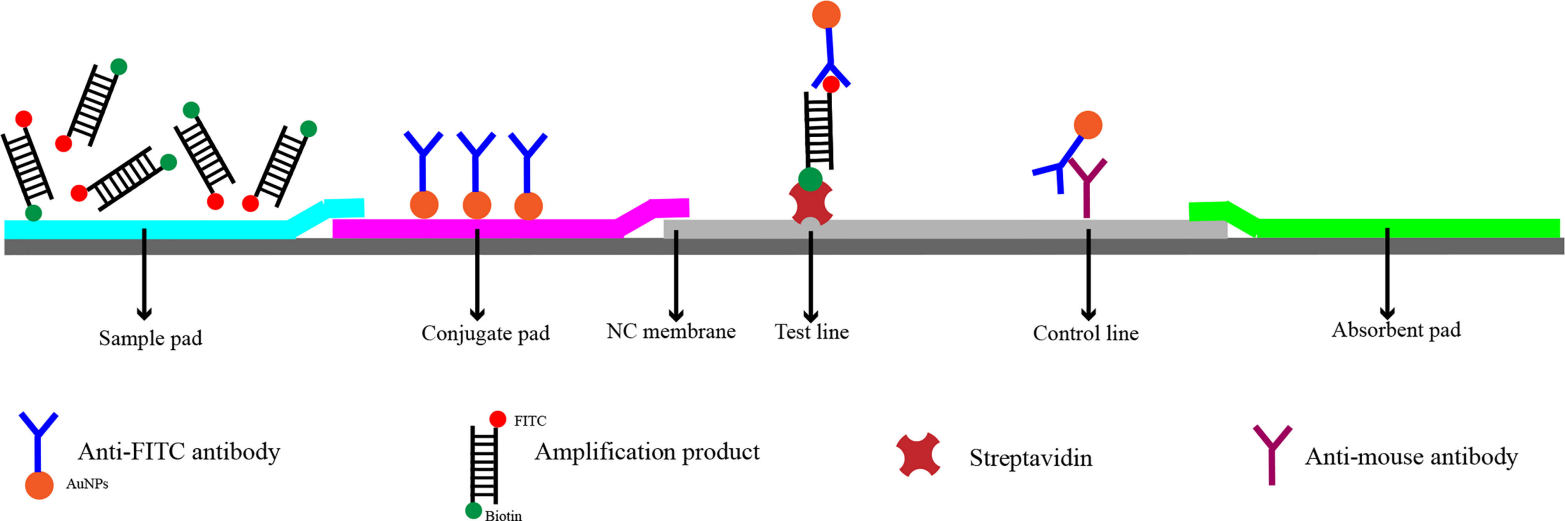
Detection principle of RPA-LFS. The shapes and their representing molecules are listed under the strip drawing.

In recent years, RPA has been widely used for the detection of pathogenic bacteria due to its rapidity, simplicity, and convenience. The RPA reaction amplifies a DNA target by enzymatically opening both strands of double-stranded DNA followed by strand-substitution activity under isothermal conditions. The DNA target shows exponential amplification within 20 min over a temperature range of 37–42°C (Liu et al., 2018). There are two main detection methods for RPA products: real-time fluorescence (using a conventional fluorescent quantitative PCR instrument) and the lateral flow strip (LFS; e.g., the colloidal gold immunochromatographic strip) (Wang et al., 2021). Use of LFSs as endpoint visual reads for amplified DNA targets makes the method less equipment dependent (Cordray and Richards-Kortum, 2015; Yongkiettrakul et al., 2017). Upstream and downstream primers are required in the RPA-LFS method, and the reverse primer is labeled with biotin at the 5’ end. It is also necessary to design a probe downstream of the forward primer that is modified by fluorescein isothiocyanate(FITC) at the 5’ end and closed by a C3 spacer at the 3’ end, with a base in the middle of the probe replaced by a tetrahydrofuran (THF, purine-pyrimidine-free site) (Li et al., 2019). When the product in the reaction system accumulates to a sufficient amount, the probe binds to the product. The nucleic acid endonuclease (*nfo*) in the reaction system then recognizes the THF site and cleaves it, exposing the 3’-OH. Strand replacement activity of *Bsu* polymerase ensures that the DNA strand after the THF site is replaced, and thus amplification occurs. Since the 5’ end of the reverse primer is labeled with biotin, the amplification product obtained from the RPA reaction is labeled with FITC at one end and biotin at the other (Wang et al., 2018) (**Figure 2**). When the diluted product is added dropwise to the sample pad, the FITC-labeled amplification product binds to anti-FITC-labeled gold nanoparticles (AuNPs) and diffuses by chromatography to the streptavidin detection line, where the biotin-labeled amplification product binds to streptavidin at one end while maintaining a positive signal through the AuNPs at the other end. Anti-FITC-labeled AuNPs that are not bound to the amplification product continue to migrate to the control line showing a red color to ensure the detection capability of the test strip (**Figure 3**).

In this study, we established a rapid and specific assay for the detection of highly virulent *K. pneumoniae*. This RPA-LFS method eliminated false-positive signals by introducing probes and base substitution. The method can be completed in 30 min at 37 °C, showing good interspecies specificity and sensitivity, and providing technical support for rapid and accurate clinical identification of highly virulent *K. pneumoniae*.

## Materials and Methods

### Ethics Statement

This study was approved by the Medical Ethics Committee of the Second People’s Hospital of Lianyungang (Permit number: 2020005). Clinical strains were collected in 2020-2021 and isolated from sputum, urine, pharyngeal swabs, drainage fluid, and other specimens. Written consent was obtained from each patient for all samples isolated.

### Bacterial Strains

Six common pathogenic bacteria, Staphylococcus aureus, Streptococcus pneumoniae, Staphylococcus epidermidis, Escherichia coli, Staphylococcus cohnii, and Pseudomonas aeruginosa, were provided by the Microbiology Department of the Second People’s Hospital of Lianyungang. 65 clinical isolates of K. pneumoniae were provided by the Second People’s Hospital of Lianyungang.

### DNA Extraction

Bacterial DNA isolation from clinical *K. pneumoniae* strains and six common pathogenic bacteria were performed using the heating and boiling method. Individual colonies were suspended in 50 μL double-distilled water (ddH_2_O), boiled for 10 min, and centrifuged at 12000 × g for 10 min; the supernatant was used as DNA template and stored at −20°C.

### Design of RPA Primers

The *rmpA2* virulence gene sequence (sequence number:MT496757) in the genome of *K. pneumoniae* was downloaded from National Center for Biotechnology Information(NCBI). The primers were designed using primer premier 5.0 software, and after entering the sequence for a specific target region, the parameters were set as follows: the product size was set to a minimum of 100 and a maximum of 500. The primer size was set to a minimum of 30 and a maximum of 35. The GC content was set to a minimum of 30% and a maximum of 70%, and all other parameters were set by default. Five pairs of primers were selected for testing.

### RPA Procedure

Recombinant enzyme polymerase amplification reactions were performed using a DNA amplification kit according to the manufacturer’s instructions (TwistDx, Maidenhead, UK). The reaction system was added in the following order: 25 µL 2× reaction buffer, 5 µL 10× Basic e-mix, 2.5 µL 20× core mix, 2.1 µL each primer (10 µM), 9.8 µL distilled water, and 1 µL template solution; 2.5 µL magnesium acetate (280 mM) was added to initiate the reaction. After brief centrifugation, the reaction mixture was immediately incubated for 30 min at 37°C. RPA amplification products were purified using a PCR wash kit (Monad Biotech Co., Ltd, Wuhan, China) and electrophoresed on a 1.5% (w/v) agarose gel.

### Design of Probes

It is known that cross-dimers formed by the probe and the reverse primer can give false positive results in RPA-LFS. Therefore, the following principles were followed when designing probes using Primer Premier 5.0 software. (1) Probe size 46–51 bp, GC content 30%–80%, and Tm 60–80°C. (2) Probe 5’ end labeled with FITC, 3’ end blocked with SpC3, and bases in the middle of the probe replaced with THF with at least 30 bp before the THF site and at least 15 bp after the THF site. (3) Use of base substitution to try to eliminate cross-dimers formed by the probe and reverse primer. (4) No modifications among the first three bases of the THF site; limited modifications to the first six bases if necessary. (5) Number of modified bases should not exceed nine bases. Primer-probe combinations were verified after base substitution for RPA false positives.

### RPA-LFS Procedure

To screen probe and primer combinations, RPA-LFS experiments were performed using the TwistAmp^®^ DNA Amplification *nfo* Kit (TwistDx). A total of 50 μL reaction system was prepared in the following order: 29.5 μL rehydration buffer, 2.1 μL forward primer, 2.1 μL reverse primer, 0.6 μL (10 μM) probe, 1 μL template solution, and 12.2 μL ddH_2_O in lyophilized powder tubes; 2.5 μL of 280 mM magnesium acetate was added to the tube cap in order to ensure the simultaneous start of all reaction systems. Immediately after transient separation, the reaction was carried out for 30 min at 37°C. RPA product (10 μL) was added to a 1.5 mL Eppendorf tube containing 190 μL ddH_2_O, and a LFS (Ubiquitous Biotechnology Co., Ltd., Hangzhou, China) was placed in the tube; results could be read after 3 min.

### Quantitative PCR

A pair of specific primers (forward: 5’-TGATTATGACATCTAAGTCTACATGCAAGG-3’; reverse: 5’-TTTACATCTGTGACACGATAGTGTTTTCTC-3’) targeting the virulence gene rmpA2 of Klebsiella pneumoniae was used for qPCR. The qPCR reaction mixture contained 10 µl of 2 × SYBR Green qPCR Mix (Tiangen Biotech Co. Ltd), 0.4 µM of each primer, 1 µl of the template, and 8.2 µl of distilled water. The cycling program was 95°C for 30 s, followed by 40 cycles of 95°C for 10 s, then 60°C for 30 s. The melting curve analysis was set as default. Cycle threshold (Ct) values less than 32 were considered as positive.

### Examination of Clinical Specimens

To evaluate the detection rate of RPA-LFS, the results were compared with those obtained by conventional PCR and qPCR, and the compliance rates of the three different assays were calculated. The PCR detection of *rmpA_2_* followed an established method reported previously (**Table 1**) (Li et al., 2020). 65 clinical strains to be tested were collected and processed using the heating and boiling method. One microliter of supernatant was used as template for RPA-LFS and PCR reactions, respectively.

**Table 1:** Primers and probes tested in this study.

Name	Sequence(5’-3’)	Length (bp)	Amplicon size (bp)
rmpA_2_-1F	TAAGTCAATGGATGTGGCTTGACATTTCGG	30	288
rmpA_2_-2F	AAATATGATAAGTCAATGGATGTGGCTTGA	30	296
rmpA_2_-3F	GATAAGTCAATGGATGTGGCTTGACATTTC	30	290
rmpA_2_-4F	TATAAGAGTATTGGTTGATAGCCGGATTTT	30	371
rmpA_2_-5F	TAAATATGATAAGTCAATGGATGTGGCTTG	30	297
rmpA_2_-R	Biotin-TTTACATCTGTGACACGATAGTGTTTTCTC	30	
rmpA_2_-P	FITC-TGATTATGACATCTATGTCAACATGCAAGG[THF]AATTTAAAAAAAACA-/C3-spacer/	45	228
rmpA_2_-mP	FITC-TGATTATGACATCTAAGTCTACATGCAAGG[THF]AATTTAATAATAACA-/C3-spacer/	30	228
PCR-F	CTGTGTCCACTATTGGTGGG	20	1046
PCR-R	GATAGTTCACCTCCTCCTCC	20	

The bases substituted in the base sequence are underlined in red.

## Results

### Screening of RPA Primers and Probes

**Figure 4:**
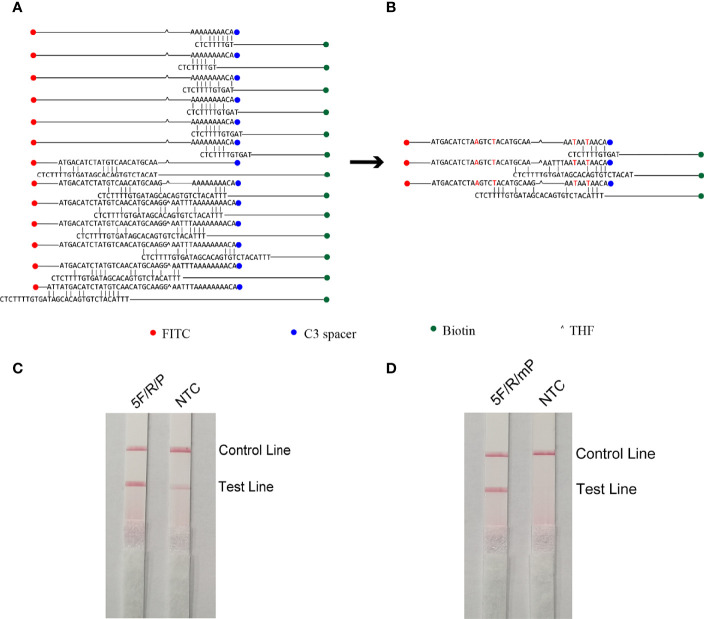
Probe design screening strategy. **(A)** Before base substitution, the probe and reverse primer form a cross-dimer. **(B)** After base substitution, the probe and reverse primer form a cross-dimer. **(C, D)** LFS results of RPA amplification products. The name of each primer-probe group is marked above the corresponding band, and the NTC lane is a template-free control for each primer-probe group.

**Figure 5:**
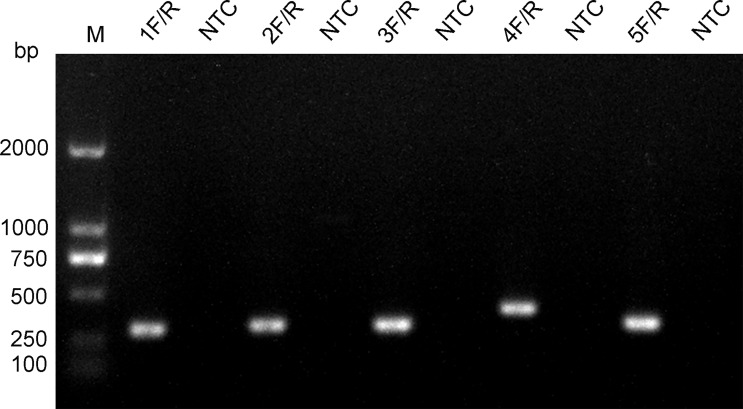
Screening of primers. The name of each primer pair is provided above each lane. The NTC lane immediately after is the no-template control of the respective primer pair. The band sizes of the DNA ladder are shown on the left.

**Figure 6:**
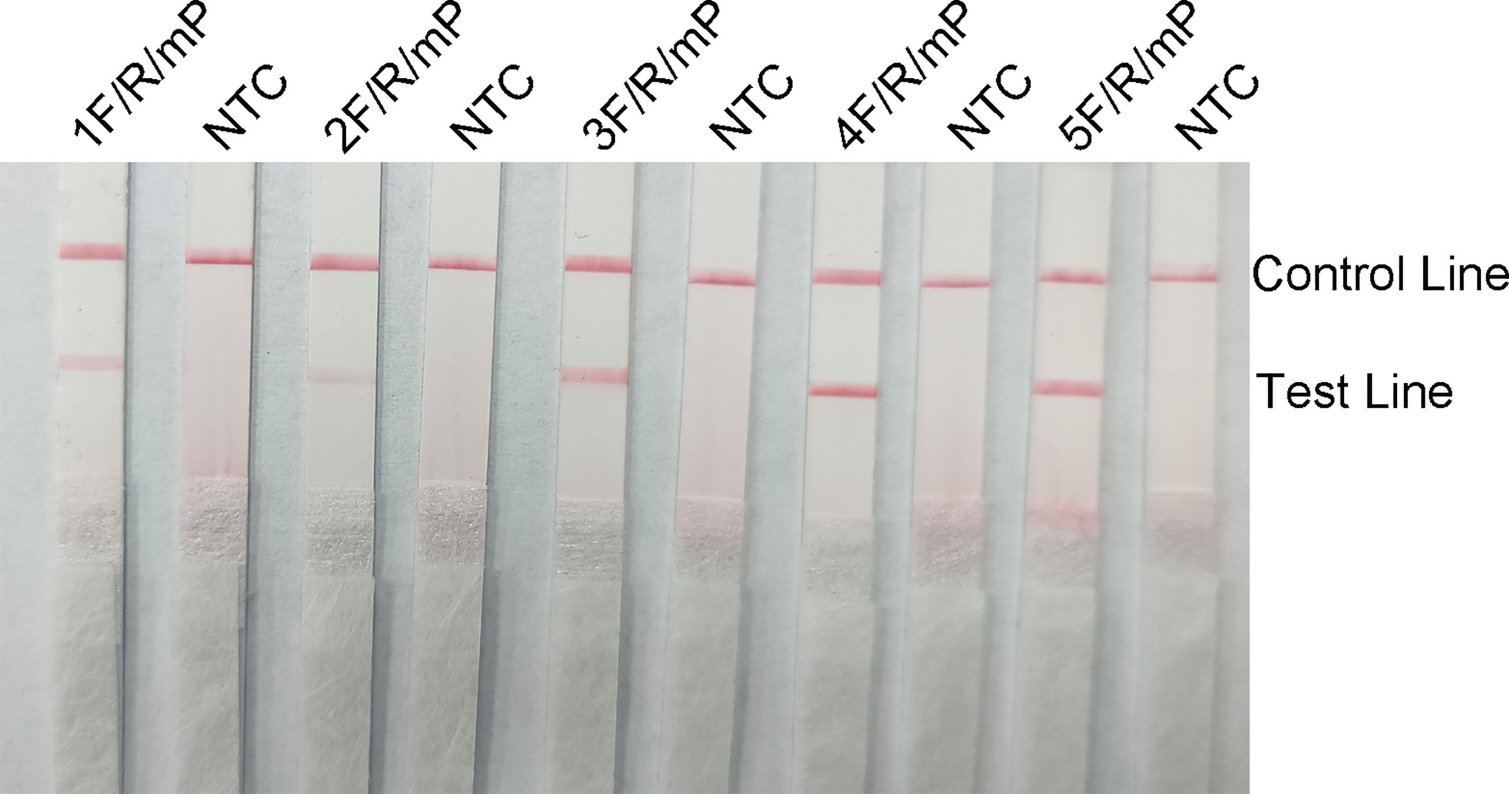
The performance of the primer-probe sets was tested using the RPA-LFS system. The LFS results of the RPA amplified products are shown in the figure. The name of each primer-probe set is labeled above the corresponding strip. The NTC strip is the template-free control for the corresponding RPA reaction on the left strip. The positions of the test and control lines are marked on the right side of the strip.

The five upstream primers designed were subjected to RPA-LFS reactions with downstream primer and probe to screen for the best upstream and downstream primer and probe combinations (**Table 1**). In designing the probes, we used primer premier 5.0 software to perform theoretical elimination of the cross-dimer formed by the probe and downstream primers to avoid false-positive signals (**Figures 4A, B**). RPA reactions were performed using genomic DNA of Klebsiella pneumoniae carrying rmpA2 as a template to verify amplification of each of the five primer pairs. As shown in the figure, all designed primers amplified the target bands as expected. The better primer pairs had brighter target bands, no non-specific amplification and had less primer dimerization (**Figure 5**). Therefore primer pair 5 was selected for subsequent detection. Firstly, we screened the probe and selected primer number 5 to perform RPA-LFS reaction with base substitution before (P) and base substitution after (mP), respectively. The results are shown in the figure, the test line of 5F/R/P showed color, but the test line also appeared colored in the condition without template, showing a false positive signal (**Figure 4C**). The primer-probe combination of 5F/R/mP showed a correct positive signal (red bands were visible in both the test and control lines), with only one red control line in the no-template control and no band in the detection line (**Figure 4D**). This indicates that the probe after base substitution eliminates the false positives. Next, we screened five primer-probe pairs and all five primer-probe combinations showed correct positive signals in the RPA-LFS test, with only one red control line in the no-template control and no bands in the test line (**Figure 6**). This indicates that none of the five primer-probe pairs showed false positives, and the primer-probe combination of 4F/R/mP had the darkest test line and therefore was the best primer-probe combination for the following RPA-LFS reactions.

### Optimization of Reaction Conditions

**Figure 7:**
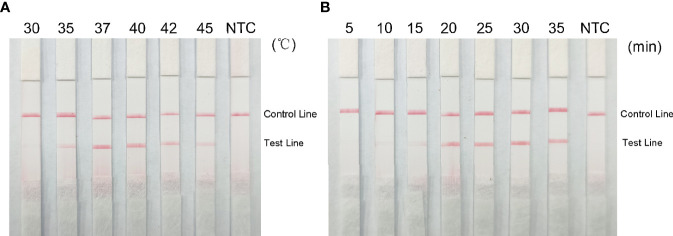
Optimal reaction temperature and time for the RPA-LFS system. **(A)** Images show the LFS results of RPA amplification at different temperatures. The temperature at which the RPA reaction was performed is shown at the top of each band. The template is the supernatant obtained from the boiling method of Klebsiella pneumoniae carrying rmpA2. The NTC strip was performed at 37°C without template control. **(B)** Images show the results of LFS for different lengths of RPA amplification. The length of time to perform the RPA reaction is shown at the top of each strip. The template is the supernatant obtained from the Klebsiella pneumoniae cooking method carrying rmpA2. The NTC strip was performed for 30 minutes without template control. The positions of the control and test lines are shown on the right side of the image.

Red bands were seen on the test line at all temperatures in the RPA-LFS analysis, most precisely at 37°C (**Figure 7A**); therefore, we chose 37°C as the optimal reaction temperature. Red stripes appeared on the test line after 5 min of reaction time and were more obvious by 25 min. The color change was not obvious after 25 min (**Figure 7B**); therefore, 25 min was chosen as the optimal reaction time.

### Specificity of the RPA-LFS Assay

**Figure 8:**
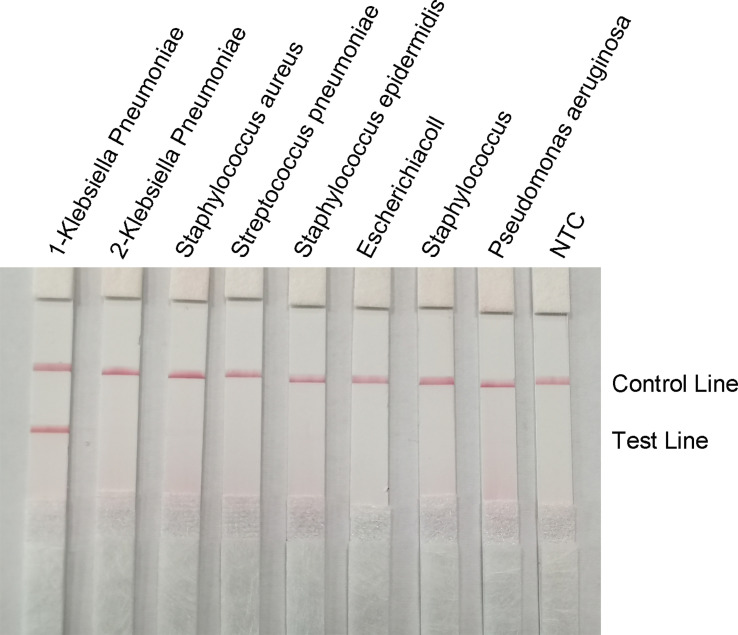
Specificity of the RPA-LFS system. The bacterial species is shown at the top of each strip. The NTC strip is a no-template control. The reaction was performed at 37°C for 30 minutes. The positions of the control and test lines are shown on the right side of the image. 1-Klebsiella pneumoniae means carrying rmpA2, 2-Klebsiella pneumoniae means not carrying rmpA2.

To confirm the specificity of the primer-probe set, RPA-LFS reactions were performed against six other common pathogens, namely *Staphylococcus aureus*, *Streptococcus pneumoniae*, *Staphylococcus epidermidis*, *E. coli*, *Staphylococcus cloacae*, and *Pseudomonas aeruginosa*. Using the genomes of these bacteria as templates did not produce a positive band, while using the genome of hvKP as a template produced a clear and specific amplification band (**Figure 8**). This suggests that the primer-probe set has good specificity for hvKP and does not cross-react with the other six pathogens and Klebsiella pneumoniae that do not carry *rmpA_2_*.

### RPA-LFS Sensitivity Assay

**Figure 9:**
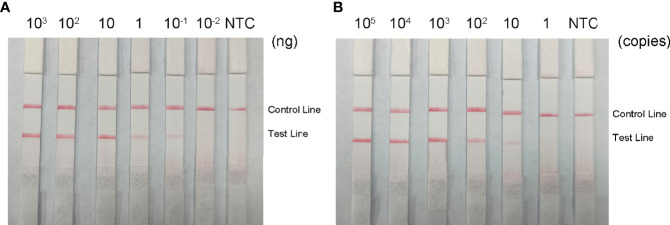
Sensitivity of the RPA-LFS system. **(A)** LFS results for different amounts of RPA carrying rmpA2 of Klebsiella pneumoniae genomic DNA. The amount added to the RPA reaction (ng/μL) is shown at the top of each band. The amplification template is the supernatant obtained from the boiling method of Klebsiella pneumoniae carrying rmpA2 **(B)** Images of the LFS results of RPA amplification using different amounts of recombinant plasmid Ss-mb-pMD™ 19-T. The amount added to the RPA reaction (in copies) is shown at the top of each strip. The NTC strip is the control without template. The positions of the control and test lines are shown on the right side of the image. Reactions were performed at 37°C for 30 minutes.

**Figure 10:**
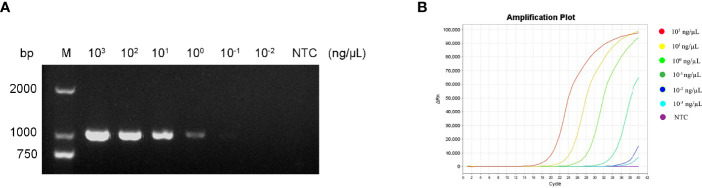
**(A)** Sensitivity of the PCR assay. **(B)** Sensitivity of the qPCR assay. The NTC strip is the control reaction without a template.

To determine the minimum detection limit of the RPA-LFS system, we diluted genomic DNA at a gradient of 10^3^–10^-2^ ng/μL. The system could detect concentrations of 10^−1^ ng/μL, and the red detection band gradually deepened as the template concentration increased (**Figure 9A**). Using genomic DNA of hvKP as a template, we amplified the *rmpA2* gene using standard PCR methods. After purifying the amplification product and ligating into the pMD 19-T vector, a positive recombinant plasmid was successfully identified by PCR and sequencing. Analysis showed 100% nucleotide sequence similarity of the recombinant plasmid with the standard sequence, which further confirmed the successful construction of recombinant plasmid Ss-mb-pMD 19-T and its suitability for subsequent experiments. The construction of the mock recombinant plasmid Ss-mb-pMD 19-T was performed using SnapGene software to obtain the full base sequence of the recombinant plasmid. The conversion of ng to copy number can then be done by selecting Tools and clicking Show DNA Calculation. DNA quantity and quality of the constructed plasmids were measured using a Qubit 9000 fluorometer, copy number was calculated, and sensitivity was measured at 10-fold dilution in a 10^5^–10^0^ copies/μL gradient. The RPA-LFS system could detect 10 copies/μL, with the red detection band gradually deepening with increasing concentration (**Figure 9B**). In addition, PCR can detect concentrations up to 1 ng/μL and qPCR can detect concentrations up to 10^-1^ ng/μL (**Figure 10**). Our established RPA-LFS was more sensitive than the PCR reaction and comparable to the sensitivity of qPCR.

### Detection of Clinical Samples

65 clinically confirmed strains of *K. pneumoniae* were examined using the RPA-LFS method 、qPCR and the conventional PCR method. 15 strains contained the virulence gene *rmpA2* and 50 strains did not contain the virulence gene *rmpA2*, with the detection results of the three methods completely consistent (**Supplementary Figure S1** and **Table 2**). However, the RPA-LFS method was more rapid and accurate with less instrument dependence.

**Table 2:** Assay performance of the RPA-LFS system and PCR.

Method	positive	negative	Time (min)
RPA-LFS	15	50	30
qPCR	15	50	120
PCR	15	50	100

## Discussion

Highly virulent *K. pneumoniae* can cause community-invasive liver abscesses and often secondary to long-distance dissemination causing brain abscesses, lung infections, and eye infections with high mortality. Rapid and accurate detection of highly virulent *K. pneumoniae* is essential for clinicians to rapidly treat patients effectively. Combining the isothermal RPA reaction with the LFS endpoint reading method, with short reaction times, low isothermal conditions, simple operation, and no instrumentation, offers a promising solution (Wu et al., 2016). This RPA-LFS technique has been successfully applied in the molecular diagnosis of diseases caused by methicillin-resistant *Staphylococcus aureus*, *Mycobacterium tuberculosis*, *Cryptococcus novelis*, and other pathogenic bacteria with exploratory significance (Yongkiettrakul et al., 2017; Ma et al., 2019; Xu et al., 2021) However, the technique places high demands on primer design, and a very small number of primer dimers may lead to false positives.

Primer-dimer formation is influenced by buffer content, ambient temperature, mixture impurities, and other factors that are difficult to avoid. Primer-dimer formation can be reduced in PCR using a hot-start strategy; however, this approach is not applicable in the RPA-LFS technique. We found that careful screening of primer pairs and avoiding consecutive matches between primer pairs could effectively prevent primer-dimer formation. To further reduce the formation of primer dimers, we introduced probes in the RPA reaction. However, the primer-probe combinations designed in this study still showed false positives.

RPA reactions can tolerate a small number of base substitutions between template and primer or probe (Daher et al., 2015; Lillis et al., 2016). It is important to note that fewer base substitution can improve the detection performance of RPA-LFS. However, we can reduce the generation of primer dimers using appropriate base substitution. First, we designed several pairs of forward and reverse primers using Primer Premier 5.0 for the virulence gene *rmpA2*. From these, we selected a pair of primers with few primer dimers predicted by the software and stretched the upstream primer by about 15 bases as the probe. Base substitution of the probe and downstream primer was performed using Primer Premier 5.0 to eliminate primer dimers. Subsequently, multiple upstream primers were designed upstream of the probe, and the best primer-probe combination was selected by combining different upstream primers with downstream primers and probes. The modified primer probes showed high sensitivity while eliminating false positive signals. The modified primer probes were specific and specifically identified highly virulent *K. pneumoniae*. Thus, we established a primer-probe combination for the detection of highly virulent *K. pneumoniae* using the RPA-LFS technique.

RPA-LFS combines the advantages of the constant temperature of RPA as well as visualization using LFS, providing a rapid and accurate detection method for highly virulent *K. pneumoniae*. When testing the primer probe specificity with other common strains, we found that it was highly specific for highly virulent *K. pneumoniae*. The lower limit of detection was as low as 10^−1^ ng/μL and 10 copies/μL. RPA-LFS was more sensitive than the PCR reaction and comparable to the sensitivity of qPCR. In fact, the amount of bacterial DNA in clinical samples was much higher than 10 copies/μL, meeting the clinical requirements for detection.

In this study, primers and probes were designed using the target sequence of the virulence gene *rmpA2*, and the results were interpreted by visual inspection only at an optimal reaction temperature of 37°C after 25 min. When used for clinical specimen testing, the sample does not need to be purified; DNA can be released by boiling and used directly for testing. The results of RPA-LFS were consistent with qPCR results and conventional PCR methods, and the detection accuracy was 100%. Thus, our RPA-LFS system provides the experimental basis for rapid detection of highly virulent *K. pneumoniae* to help clinicians take more accurate and rapid therapeutic measures.

## Data Availability Statement

The original contributions presented in the study are included in the article/**Supplementary Material**. Further inquiries can be directed to the corresponding authors.

## Ethics Statement

This study was approved by the Medical Ethics Committee of the Second People’s Hospital of Lianyungang (Permit number: 2020005). Clinical strains were collected in 2020-2021 and isolated from sputum, urine, pharyngeal swabs, drainage fluid, and other specimens. Written consent was obtained from each patient for all samples isolated.

## Author Contributions

NL, FM and WL conceived and designed the experiments and wrote the paper. LW, FW and HC performed the experiments. ST, QZ and LL analyzed the data. All authors discussed the results, provided comments and approved the final version of the manuscript.

## Funding

The work was funded by the Natural Science Foundation of Jiangsu Province (BK20191210), the “Project 333” training fund of Jiangsu Province (BRA2019248), Lianyungang City “521 Project” project (LYG06521202160) and Postgraduate Research Innovation Program of Bengbu Medical College (Byycxz21007).

## Conflict of Interest

The authors declare that the research was conducted in the absence of any commercial or financial relationships that could be construed as a potential conflict of interest.

## Publisher’s Note

All claims expressed in this article are solely those of the authors and do not necessarily represent those of their affiliated organizations, or those of the publisher, the editors and the reviewers. Any product that may be evaluated in this article, or claim that may be made by its manufacturer, is not guaranteed or endorsed by the publisher.

## Supplementary Material

Supplementary Figure 1 **(A)** qPCR results of clinical samples. **(B)** PCR results of clinical samples.
