# Supplementary figures and images for: Rapid Detection of Klebsiella pneumoniae Carrying Virulence Gene rmpA2 by Recombinase Polymerase Amplification Combined With Lateral Flow Strips
Source: Front Cell Infect Microbiol. 2022 May 19;12:877649. doi: 10.3389/fcimb.2022.877649 (PMC9160666; doi:10.3389/fcimb.2022.877649)

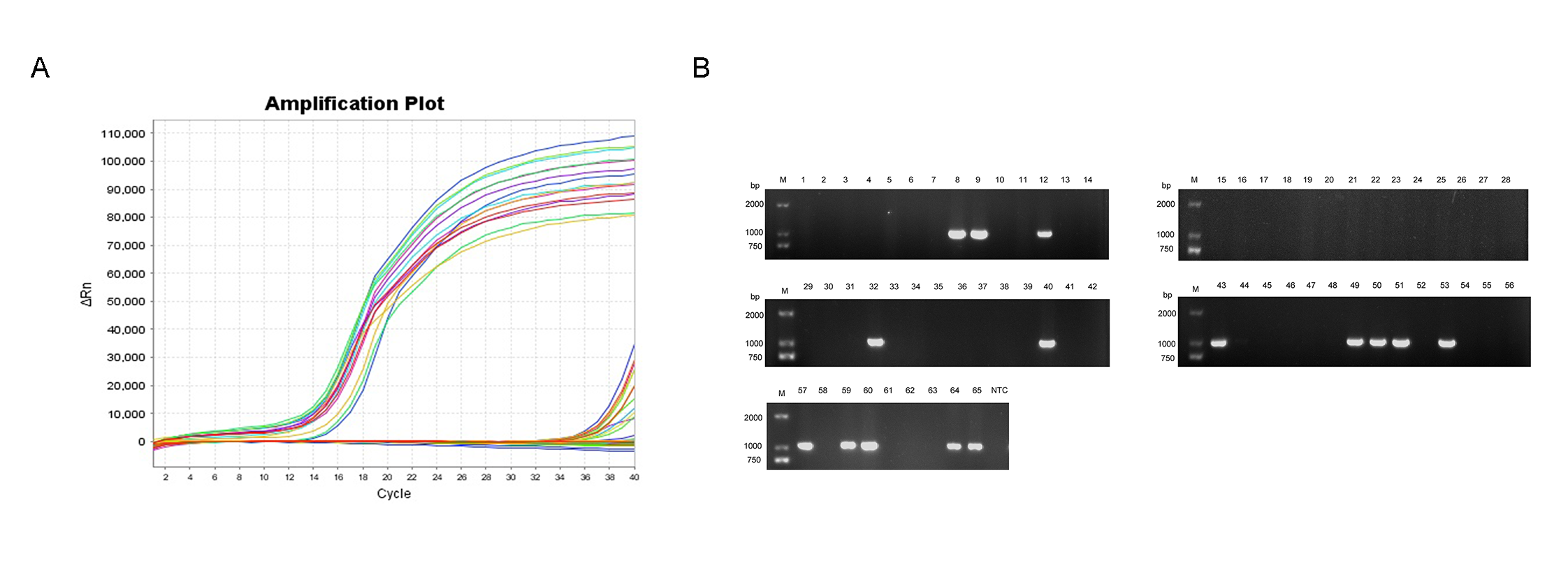

Supplement: Supplementary Figure 1 — (A) qPCR results of clinical samples. (B) PCR results of clinical samples. [file Image_1.tif]
